# Supplementary material for: Organelle partitioning in the multi-budding yeast Aureobasidium pullulans
Source: bioRxiv. 2026 Apr 21:2026.04.17.719237. Preprint. [Version 1] doi: 10.64898/2026.04.17.719237 (PMC13131616; doi:10.64898/2026.04.17.719237)
Supplement: 1 [file NIHPP2026.04.17.719237V1-supplement-1.pdf]

bioRxiv preprint doi: https://doi.org/10.64898/2026.04.17.719237; this version posted April 21, 2026. The copyright holder for this preprint (which was not certified by peer review) is the author/funder, who has granted bioRxiv a license to display the preprint in perpetuity. It is made available under aCC-BY 4.0 International license.

Supplemental Figure 1

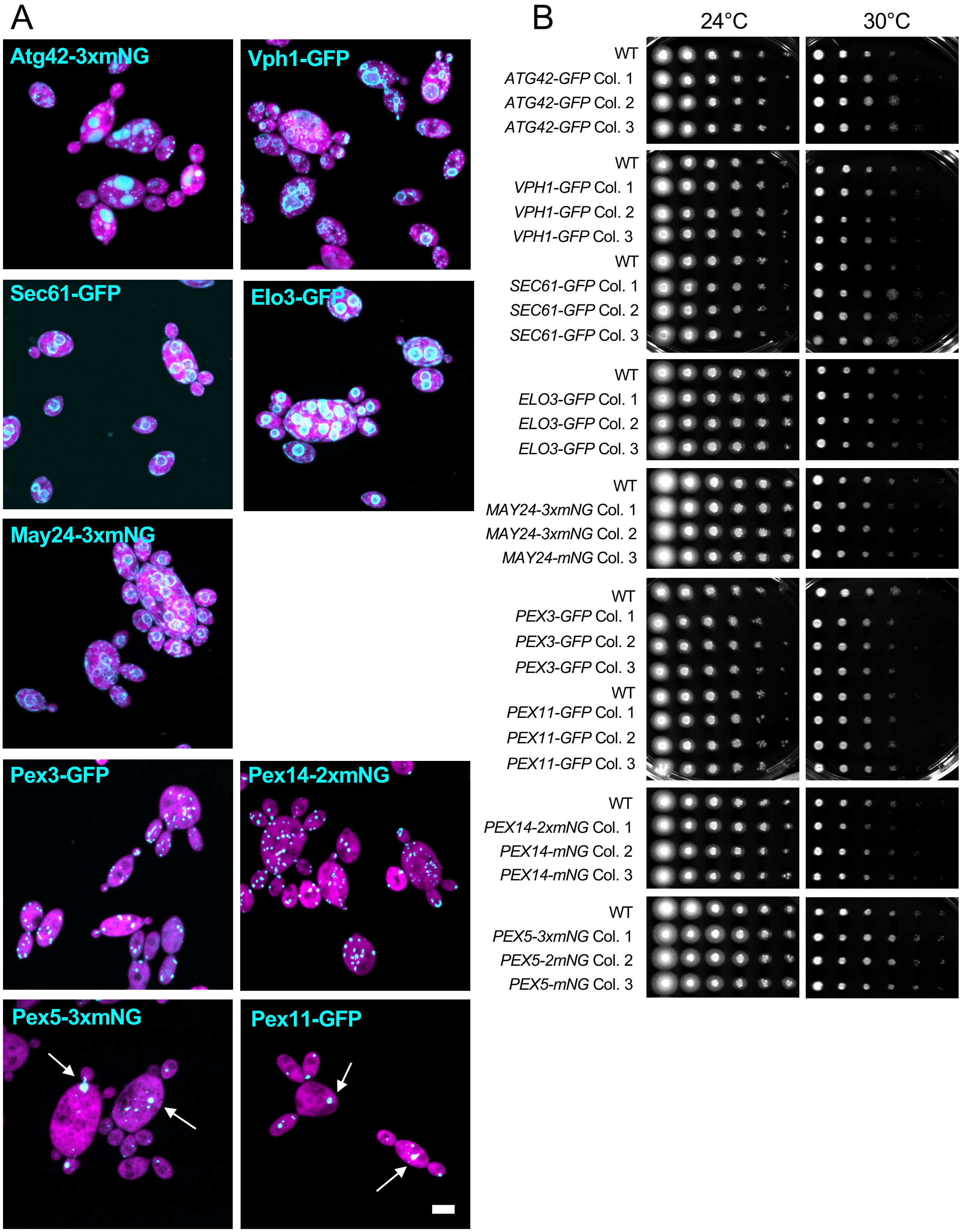

bioRxiv preprint doi: https://doi.org/10.64898/2026.04.17.719237; this version posted April 21, 2026. The copyright holder for this preprint (which was not certified by peer review) is the author/funder, who has granted bioRxiv a license to display the preprint in perpetuity. It is made available under aCC-BY 4.0 International license.

Supplemental Figure 1: **Characteriztion of organelle markers in *A. pullulans*.** (**A**) Maximum intensity projections of confocal Z-series showing *A. pullulans* cells expressing a cytosol marker (3xmCherry, magenta) and each indicated organelle marker (cyan) tagged with GFP or mNG. The vacuole lumen was visualized using Atg42-3xmNG (DLY25963) and the vacuole membrane was visualized using Vph1-GFP (DLY24947). The ER was visualized using Sec61-GFP (DLY24944), Elo3-GFP (DLY27372), and May24-3xmNG (DLY27375). Peroxisomes were visualized using Pex3-GFP (DLY25001), Pex14-2xmNG (DLY27366), Pex11-GFP (DLY25004), and Pex5-3xmNG (DLY27370). Expression of Pex5-mNG or Pex11-GFP resulted in large clumps of peroxisomes (white arrows). Scale bar, 5 µm. (**B**) Growth assays on YPD showing 10-fold serial dilutions of three separate transformants with the indicated genotypes. Plates were grown for 48 h at the indicated temperatures.

bioRxiv preprint doi: https://doi.org/10.64898/2026.04.17.719237; this version posted April 21, 2026. The copyright holder for this preprint (which was not certified by peer review) is the author/funder, who has granted bioRxiv a license to display the preprint in perpetuity. It is made available under aCC-BY 4.0 International license.

Supplemental Figure 2

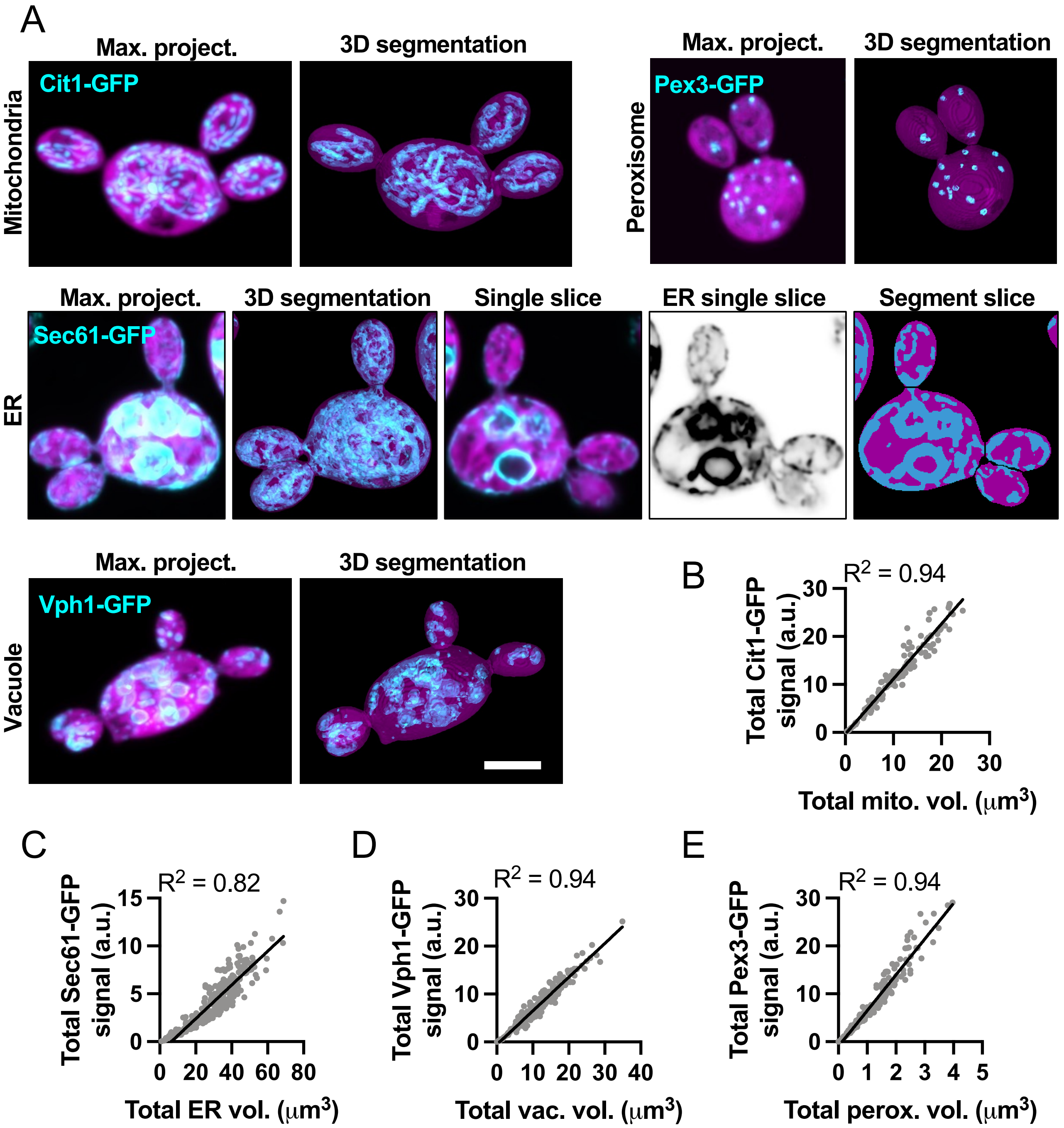

Supplemental Figure 2: **Quantification of organelle content.** (**A**) Maximum intensity projections of confocal Z-series showing *A. pullulans* cells expressing a cytosol marker (3xmCherry, magenta) and different organelle markers in cyan: Cit1-GFP (DLY25312), Sec61-GFP (DLY24944), Vph1-GFP (DLY24947), and Pex3-GFP (DLY25001), labeling the mitochondria, ER, vacuole, and peroxisomes, respectively. To the right of each image we show the 3D segmentation used to measure cell and organelle volume. For the ER we also include a cross section to highlight cortical and internal ER structures. Scale bar, 5 μm. (**B-E**) Correlation between total organelle volume and total GFP signal measured in each bud for cells expressing different organelle markers: (**B**) mitochondria, (**C**) ER, (**D**) vacuole, and (**E**) peroxisome. R^2 values are shown (n = 154, 304, 225, and 249 buds, respectively).

bioRxiv preprint doi: https://doi.org/10.64898/2026.04.17.719237; this version posted April 21, 2026. The copyright holder for this preprint (which was not certified by peer review) is the author/funder, who has granted bioRxiv a license to display the preprint in perpetuity. It is made available under aCC-BY 4.0 International license.

Supplemental Table 1

Strain Name	Relevant Genotype
DLY24944	*URA3:3xmCherry; SEC61-GFP:HYG* ^{R}
DLY24947	*URA3:3xmCherry; VPH1-GFP:HYG* ^{R}
DLY25001	*URA3:3xmCherry; PEX3-GFP:HYG* ^{R}
DLY25004	*URA3:3xmCherry; PEX11-GFP:HYG* ^{R}
DLY25312	*URA3:3xmCherry; CIT1-GFP:NAT* ^{R}
DLY25963	*URA3:3xmCherry; ATG42-3xmNG:HYG* ^{R}
DLY26335	*URA3:3xmCherry; VPH1-GFP:HYG* ^{R} *; pak1ΔNAT* ^{R}
DLY26338	*URA3:3xmCherry; PEX3-GFP:HYG* ^{R} *; pak1ΔNAT* ^{R}
DLY26341	*URA3:3xmCherry; SEC61-GFP:HYG* ^{R} *; pak1ΔNAT* ^{R}
DLY26344	*URA3:3xmCherry; CIT1-GFP:NAT* ^{R} *; pak1ΔHYG* ^{R}
DLY26775	*SEC61-3xmCherry:HYG* ^{R} *; VPH1-3xmNG:NAT* ^{R}
DLY27366	*URA3:3xmCherry; PEX14-2xmNG:NAT* ^{R}
DLY27370	*URA3:3xmCherry; PEX5-3xmNG:NAT* ^{R}
DLY27372	*URA3:3xmCherry; ELO3-GFP:NAT* ^{R}
DLY27375	*URA3:3xmCherry; MAY24-3xmNG:NAT* ^{R}
